# Pyrexia of Unknown Origin: An Atypical Presentation of Hepatic Hemangioma

**DOI:** 10.1055/s-0040-1721428

**Published:** 2020-12-14

**Authors:** Gunjan Desai, Dattaraj Budkule, Prasad Pande, Prasad Wagle

**Affiliations:** 1 Department of Surgical Gastroenterology, MGM New Bombay Hospital, Navi Mumbai, Maharashtra, India; 2 Department of General Surgery, Lilavati Hospital and Research Centre, Mumbai, Maharashtra, India; 3 Department of Surgical Gastroenterology, Lilavati Hospital, Mumbai, Maharashtra, India

**Keywords:** liver tumor, transarterial embolization, liver resection

## Abstract

Pyrexia of unknown origin (PUO) has been a diagnostic challenge for decades. Hepatic hemangioma (HH) is not a common differential diagnosis of PUO. It is the most common benign hepatic tumor, commonly asymptomatic and incidentally detected, or can present with vague abdominal pain. PUO is a rare presenting feature. We describe a case of 38-year-old lady presenting with PUO. With no other identifiable source of fever despite exhaustive investigations, a giant hemangioma in right lobe of liver detected on abdominal ultrasonography was deemed to be the cause of PUO. The patient had sudden decrease in hemoglobin while undergoing workup, which on imaging showed a bleeding hemangioma and right hepatectomy was performed. Patient had an uneventful recovery and her PUO also resolved after surgery. HH should be considered a rare diagnosis of exclusion for PUO after a standard algorithmic approach does not reveal any other cause.

Pyrexia of unknown origin (PUO) has perplexed clinicians since its first description in 1961 by Petersdorf and Beeson. They defined PUO as fever above 38°C or 101°F for longer than 3 weeks without an established diagnosis after a week of inpatient investigations. 1 Over years, its causes have been identified as infective, of which tuberculosis is the most common cause in India, inflammatory, malignant, rare causes such as Kikuchi disease, adrenal insufficiency, biliary cholangitis, and finally idiopathic, which accounts for up to 1.6% cases. 2 Hepatic hemangioma (HH) is a very rare cause of PUO with only a few case reports describing this association. It is not routinely considered in the differential diagnosis of PUO. 3 4 5

HH is the most common benign hepatic tumor. 6 It is usually asymptomatic, being incidentally diagnosed, or may present with pain and/or compressive symptoms. PUO is a rare presenting feature of HH. 3 4 5 We present a case of giant HH presenting as PUO, describe its management, and review the literature in brief.

## Case Report

A 38 years old lady with no comorbidities presented to the surgical gastroenterology department of a tertiary care center with intermittent low-grade fever without chills or rigors for 1 month. It was associated with right upper abdominal discomfort. She had no other symptoms, no history of travel, contact with pets; she had no tattoos and no history of recreational drug use, alcohol, or smoking. She was hemodynamically stable with nontender hepatomegaly 4 cm below the right costal margin. Rest of the abdominal and general examination was normal. Respiratory, cardiovascular, and central nervous system examination was also normal.

Complete hemogram, liver, and renal function tests were normal. Erythrocyte sedimentation rate was 52 mm/h (normal—up to 32 mm/h) and C-reactive protein was 12 mg/L (normal up to 5 mg/L). She was worked up exhaustively and meticulously for PUO and infective, inflammatory, and autoimmune etiologies were ruled out.

**Fig. 1:**
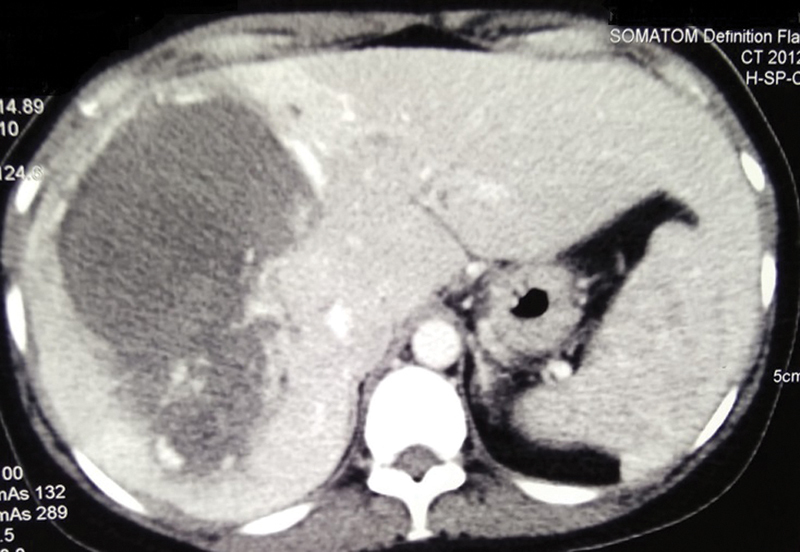
Computed tomography scan arterial phase depicting large peripherally enhancing lesion involving the right lobe of liver.

During this period, her hemoglobin dropped from 12.6 g/dL to 9 g/dL, without any hemodynamic instability or overt signs of bleeding. Abdominal ultrasonography (USG) showed a large hyperechoic lesion in the right lobe of liver with internal vascularity. Contrast-enhanced computed tomography of the abdomen showed a large hypodense lesion involving hepatic segment 5, 6, 7, and 8 with areas of hemorrhage and thrombus in the lesion in noncontrast phase with centripetal contrast enhancement in arterial phase and delayed washout with contrast pooling in the lesion ( Fig. 1) . A positron emission tomography scan was also performed that did not reveal any focus of infection.

**Fig. 2:**
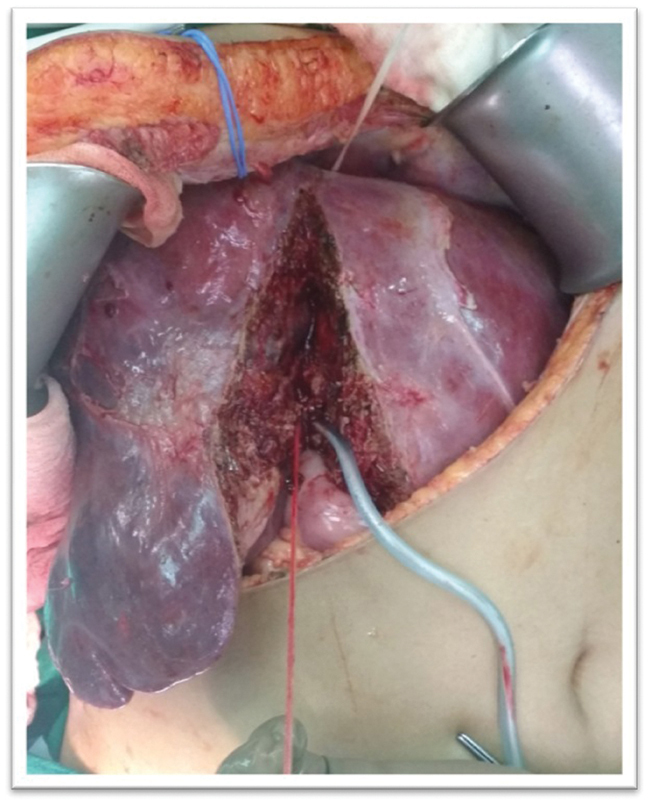
Intraoperative image depicting hanging maneuver for hepatectomy for large hepatic lesions.

**Fig. 3:**
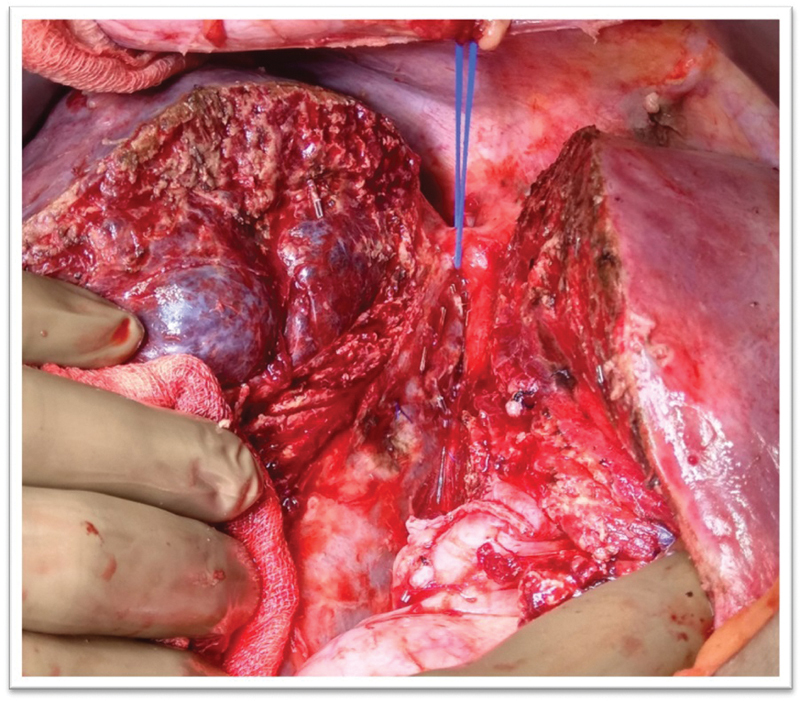
Intraoperative image depicting cut surface of liver with intact hemangioma with the specimen hanging on the right hepatic vein and middle hepatic vein on the cut surface of future liver remnant.

In view of symptomatic giant HH and suspected hemorrhage, right hepatectomy was planned, with functional liver remnant of 40%. Preoperative bland arterial embolization was done 1 day prior to surgery. Right hepatectomy was performed with intermittent selective right Glissonian pedicle occlusion and a combination of posterior and anterior hanging approach ( Figs. 2 , 3 ). The procedure was uneventful with a blood loss of 300 mL.

The patient recovered uneventfully and was afebrile after surgery. She was discharged on postoperative day 6. Histopathology revealed a cavernous hemangioma with areas of thrombosis and hemorrhage. The patient is asymptomatic at 9 months follow-up.

## Discussion

**Fig. 4:**
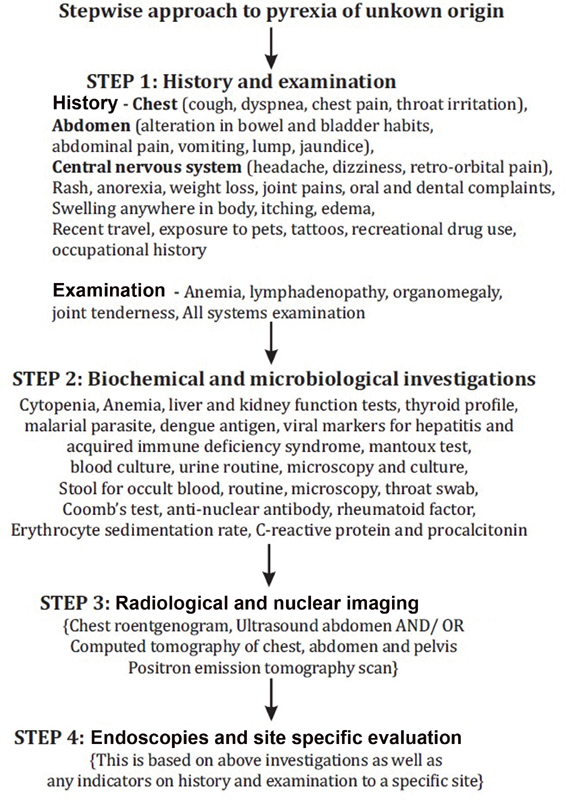
Our stepwise approach to diagnosis of pyrexia of unknown origin.

PUO has been a diagnostic challenge for decades. There have been frequent changes in its definition, especially with regard to the temporal criteria, which has changed from 1 week of inpatient investigation in its first definition to 3 days of outpatient or inpatient investigations by Durack and Street to the latest defined “pragmatic investigation period” by Knockaert et al and structured diagnostic checklist without any time frame. 1 7 8 Across the literature, the key aspect is presence of a structured diagnostic algorithm for cases with PUO to reduce the number of undiagnosed and idiopathic cases. Our stepwise approach to PUO is highlighted in Fig. 4 . Giant HH as a cause of PUO is a diagnosis of exclusion as established in our case after thorough and exhaustive attempt to rule out common causes of PUO.

HH is the most common benign hepatic tumor, constituting ∼70% of them. 4 5 HHs are blood-filled cavities within the liver parenchyma lined by endothelial cells fed by a branch of hepatic artery. 5 6 The incidence of HH is reported to be 0.4 to 20%. 4 5 9 They have female predilection 5:1. The mean age of presentation is 30 to 50 years. 4 9 They vary in size from few millimeters to 3 cm (small), 3 to 10 cm (medium), and more than 10 cm (giant hemangiomas). 6 The exact pathophysiology is poorly understood and congenital factors, abnormal vasculogenesis, and hormonal factors (estrogen dependence) play some role. 4 10

HHs are usually small, solitary, in the right lobe and most commonly incidentally detected. Less common symptoms are vague abdominal discomfort, right upper quadrant pain, anorexia, nausea, vomiting, and, rarely, PUO. 4 10 11 HH can present with complications such as compression (gastric outlet obstruction, jaundice), rupture, bleeding or coagulopathy. (Kasabach–Merritt syndrome due to exposure of platelets to the subendothelial collagen in hemangiomas leads to their activation and consumption, causing thrombocytopenia and purpuras.). 12

The initial diagnostic modality is usually USG that shows a homogenous hyperechoic lesion with sharp margins and acoustic enhancement. 4 However, inhomogenous lesions with mixed echogenicity can be seen with fibrosis, thrombosis, or hemorrhage. 6 USG with Doppler shows no perilesional vascularity unlike in hepatocellular carcinomas and metastatic lesions. On liver protocol computed tomography, HH appears as a well-defined hypodense lesion with peripheral nodular enhancement with progressive centripetal homogenous contrast filling with slow washout, unlike that in malignant lesion where there is a rapid washout. 5 6

Magnetic resonance imaging with diagnostic sensitivity and specificity of more than 90% shows characteristic well-demarcated homogenous lesion, hypointense in T1-weighted images, and hyperintense on T2-weighted images, with persistent contrast enhancement on delayed phase imaging. 6 Technetium 99-labeled red blood cell pool scintigraphy is 100% specific, which shows perfusion/blood pool mismatch, that is, decreased perfusion on early dynamic images and a gradual increase in activity on blood pool images over time. Biopsy is not performed due to high risk of hemorrhage as well as its low yield. 11 12

Small HH can be managed conservatively. Surgery is considered in cases of rapid increase in size, intractable pain, giant HH or complications such as rupture, bleeding, or Kasabach–Merritt syndrome. 6 12 In a study, persistent abdominal symptoms were the most common indication for surgery. 13 A nationwide survey in Japan, in patients with ≤5 cm hemangioma, surgical resection was indicated when a malignant tumor cannot be ruled out. 14 Surgical options include enucleation and formal hepatectomy. Preoperative embolization helps decrease the size of the lesion and reduces intraoperative blood loss. 9 Surgery can be combined with percutaneous or laparoscopic radiofrequency ablation. Nonsurgical options include radiotherapy, usually 15 to 30 Gray, 15 to 22 fractions over 3 weeks. Some role of chemotherapy, although controversial, has also been demonstrated with bleomycin, etoposide, cisplatin. 5 9 In case of giant hemangiomas, or hemangiomas involving both hepatic lobes, liver transplant can be considered. 6

Table 1 summarizes the recent literature of HH presenting as PUO. 9 11 15 16 17 This atypical presentation of HH was first described by Schumacker in 1942, where one out of 66 surgically treated liver hemangioma patients had fever. 18 Although not certain, Kupffer cells are believed to be the source of endogenous pyrogens that can cause persistent fever in HH. A case of PUO due to giant HH managed successfully with right hepatectomy is described. 9 In contrast, another report described successful management of HH presenting as PUO with conservative management. 11

**Table 1:** Brief review of cases of hepatic hemangioma presenting with pyrexia of unknown origin reported in the literature and their comparison with our case

Number	Authors	Age	Sex	Symptoms	Duration	Treatment
1	Kathleen Hopkins 9	46	Female	Right upper quadrant abdominal pain, fever, chills, night swears, anorexia, cough	1 mo	Right hepatectomy
2	Lee et al 11	37	Female	Fever	15 d	Observation
3	van Gorcum et al 15	41	Female	Fever, night sweats, malaise, pain, weight loss	–	Surgical excision
4	Pandit et al 16	49	Female	Fever	3 mo	Laparoscopic-assisted left lateral segmentectomy
5	Liu et al 17	33	Male	Fever	2 mo	Right trisectionectomy
6	Our case	30	Female	Fever	1 mo	Right hepatectomy

## Conclusion

PUO needs a stepwise algorithmic approach to diagnosis and institutional protocols would be helpful to take into consideration the demographic conditions affecting the diagnosis. HH as a cause of PUO is a diagnosis of exclusion as these are rarely symptomatic even when they are giant and should be considered cause of PUO after exhaustive attempts to rule out other causes. Surgical excision needs to be considered as a treatment of PUO in these cases.
